# Prophylactic and immune modulatory influences of *Nigella sativa* Linn. in broilers exposed to biological challenge

**DOI:** 10.14202/vetworld.2017.1447-1455

**Published:** 2017-12-12

**Authors:** Essam S. Soliman, Rania T. Hamad, Amira Ahmed

**Affiliations:** 1 Department of Animal Hygiene, Zoonosis and Animal Behavior, Faculty of Veterinary Medicine, Suez Canal University, Ismailia 41522, Egypt; 2 Department of Pathology, Faculty of Veterinary Medicine, Suez Canal University, Ismailia 41522, Egypt; 3 Department of Poultry and Rabbit Medicine, Faculty of Veterinary Medicine, Suez Canal University, Ismailia 41522, Egypt

**Keywords:** Broiler, *Escherichia coli*, histopathology, *Nigella sativa* Linn, preventive

## Abstract

**Background and Aim:**

Prophylaxis and disease prevention is an essential strategy among biorisk management in poultry farms that stimulate and maintain the birds’ immunity. The aim of this study was to investigate the prophylactic, and immune-stimulant influence of *Nigella sativa* Linn. in broilers under biological stress.

**Materials and Methods:**

A total of 250 1-day-old (ross) chicks were divided into 5 groups; four of which were supplemented with 1.4%, 2.8%, 4.2%, and 5.6% *N. sativa* Linn., respectively. The four supplemented groups were challenged with *Escherichia coli* O_157_:H_7_ 1.5×10^8^ at a 14^th^ day old. A total of 1050 samples (150 serum, 150 swab, and 750 organ samples) were collected and examined.

**Results:**

A highly significant increase (p<0.01) in 5.6% *N. sativa* Linn. supplemented group in performance traits (body weight, weight gain, and performance index), biochemical parameters (proteinogram, liver enzymes, and creatinine), immunoglobulins concentration, and immune organs’ weight. Meanwile, liver showed improvement of histoarchitecture without fibrosis. Heart showed a mild pericarditis with a mild degree of hydropic degeneration. Bursa, thymus, and spleen showed lymphoid hyperplasia.

**Conclusion:**

A concentration of 5.6% *N. sativa* Linn. in broiler’s feed can improve the immune response and subsequent resistance of broilers against diseases.

## Introduction

The world’s direction in the past decades was focusing on the usage of growth promoters for improving the productive performance; therefore, antibiotics were used extensively in poultry industry as growth promoters [1]. The results of using antibiotics were magnificent in increasing the performance traits, but they contributed the development of antibiotics-resistant bacteria [2,3]. A second brand of growth promoters was developed and used; prebiotics and probiotics that contributed enhancement in the intestinal mucosa with a significant increase in performance and resistance of bird through acting as competitive exclusive for pathogenic organisms [4-6]. Although from an economic point of view, using these products on a daily basis makes their use unpractical and expensive for the producer.

There is a great interest in developing natural alternatives for the growth promoters. *Nigella sativa* Linn. and its oil have been widely used for centuries in the treatment of different physiological disorders [1]. It has been considered one of the greatest forms of healing medicine available, especially in Middle East and Asia, as it was mentioned that black seed is the remedy for all diseases except death in one of the prophetic hadith. It is also recommended for use on regular basis in Tibb-e-Nabwi (prophetic medicine) [7]. The nutritional values of N. sativa meal were documented in the study of Attia *et al*. [8,9] and El-Deek *et al*. [10]. *N. sativa* Linn. seeds, its oil, extracts, and some of its active principles, particularly thymoquinone and alpha-hederin, possess remarkable *in vitro* and *in vivo* pharmacological activities against a large variety of diseases and found to be relatively safe [11,12].

The aim of this study was to investigate the prophylactic, and immune-stimulant influence of *N. sativa* Linn. in broilers under biological stress (*Escherichia coli* O_157_:H_7_ 1.5×10^8^ at the 14^th^ day old).

## Materials and Methods

### Ethical approval

All applicable international, national, and/or institutional guidelines for the care and use of birds were followed. Staff managed and treated birds as sentient and regarded their proper care and use with minimization of discomfort, distress, and pain. Birds’ numbers were minimized to obtain scientifically valid results and to minimize the great suffering of birds. The experiment was not repeated unnecessarily. Birds were housed, feed, and handled in accordance to their needs (behavioral and biological).

### Experimental design

A total of 250 one-day-old chicks (ross) were purchased and housed on battery system. The chicks were divided into five groups; each group consisted of 50 chicks (five replicates of ten birds). The birds were brooded at 33°C with a gradual decline by 3°C weekly until 25°C by the end of 3^rd^ week. The room was supported with natural ventilation means. Artificial light was supplied for at least 18 h a day. Birds were given *ad libitum* access to water, as well as a standard corn-soybean basal diet was supplied to meet broiler’s dietary requirements as tabulated in Table-1 according to NRC [13].

**Table-1:** Feed ingredient and nutrient composition of basal ration at different growth stages of experimental birds.

Components	Starter ration 1:10 days (%)	Grower ration 11:38 days (%)
Food ingredient		
Yellow corn	54	55
Soybean meal	22	16
Corn gluten	10	15
Lime	0.50	0.50
Dicalcium phosphate	1.0	1.0
Sodium chloride	0.5	0.5
DL. Methionine	2.0	1.0
L. lysine	1.50	1.50
Soybean oil	3.5	4.5
Vitamin premix	2.5	2.5
Mineral premix	2.5	2.5
Nutrient content		
Energy	2990 Kcal/kg	3000 Kcal/kg
Protein	23%	22.5%
Fat	5.60%	2.84%
Row fiber	3.80%	3.39%

All birds were vaccinated in drinking water with infectious bronchitis vaccine (live attenuated virus of IB-H120 ≥10^3.5^ EID_50_/dose) at the 7^th^ day of age, infectious bursal disease vaccine (live attenuated virus of VMG91 ≥10^3.0^ TCID_50_) at the 14^th^ and 21^st^ days of age, and Newcastle disease virus vaccine (live lentogenic ND virus of Lasota ≥10^6.0^ EID_50_) at the 18^th^ and 28^th^ day. The experiment was designed to last for about 5 weeks (38 days). Mortalities were noticed and recorded daily. Indoor temperature and relative humidity were monitored and recorded daily during the experiment.

### N. sativa Linn.

*N. sativa* Linn. crushed seeds were purchased from authenticated market shop, grinded, and added to the ration from the 1^st^ day of age by rates of 1.4% (1.4 g/100 g ration) for Group 1 (G1), 2.8% (2.8 g/100 g ration) for Group 2 (G2), 4.2% (4.2 g/100 g ration) for Group 3 (G3), and 5.6% (5.6 g/100 g ration) for Group 4 (G4). The fifth group was kept as control group.

### *E. coli* challenge

Four of the five groups (G1, G2, G3, and G4) were challenged with *E. coli* O_157_:H_7_ 1.5×10^8^ at the 14^th^ day of age in drinking water [14].

### Performance indices

Average live body weight (LBW) was estimated by weighting representative samples of birds on a weekly intervals (a total of 150 birds were weighted weekly; about 30 birds per group, the birds were chosen randomly and representative for each group), as well as the weekly feed intake, and the amount consumed by each bird per grams was calculated based on the birds capacity. Based on the estimated LBW and the calculated feed intake, a number of indices were calculated as indicators for broiler performance including: Weekly body weight gain (BWG) calculated by subtracting X1 LBW from X2 LBW and expressed as grams/week, weekly feed conversion ratio (FCR) calculated by dividing weekly feed intake per grams to weekly BWG per gram, and weekly performance index (PI) calculated by dividing average weekly LBW by kg to weekly FCR [15].

### Sampling

A total of 1050 samples (150 serum, 150 swab, and 750 organ samples including thymus, spleen, bursa, heart, and liver) were collected during the study period, and sampling was carried out on a weekly basis (six birds/each group/week). Blood samples were collected from the five groups and kept for overnight at 4°C, centrifuged at 3000 rpm for 20 min for serum separation. A clear non-hemolyzed sera were divided into 3 equal parts in Eppendorf tubes, stored at −20°C until used for biochemical analysis [16]. Birds (about 30 birds/week) were slaughtered after blood sampling; thymus, spleen, bursa, heart, and liver were removed, weighed, and expressed as g/kg BW, and then, all organs were washed with 5% phosphate buffered saline (PBS) and kept on 10% buffered formalin for histopathological examination. Swab samples were collected from birds’ intestine on 9 mL phosphate buffer saline, preserved in the icebox, and transferred to the laboratory for bacteriological assessment.

### Biochemical analysis

Serum samples were examined for the biochemical changes in some parameters as total protein, albumin, globulin, alanine aminotransferase (ALT), aspartate aminotransferase (AST), urea, and creatinine calorimetrically [17]. Serum immunoglobulin G (IgG), IgM, and IgA concentrations were measured using immunoturbidimetric assay [18].

### Bacteriological examination

All swabs were prepared according to APHA [19]. On arrival to the laboratory, 10-fold serial dilution was carried out up to 10^−6^ to cover the expected range of samples contamination, which could be easily counted.

Bacterial counts (total bacterial count [TBC] and total *Enterobacteriaceae* count [TEC]) were applied using drop plate technique [20]. TBC was performed using standard plate count agar at 37°C for 24-48 h. On the other hand, TEC was conducted using eosin methylene blue agar at 37°C for 24-48 h. Plates showed 30-300 CFU per plate were counted [21].

### Histopathological examination

Thymus, spleen, bursa, heart, and liver were washed with 5% PBS, preserved in 10% buffered formalin saline until further processing. Specimens were cut into 5 mm thickness sections and put into tissue cassettes. They were dehydrated by transferring through a series of alcohols with increasing concentrations, cleared in xylol, and embedded in paraffin. The obtained sections were stained with hematoxylin and eosin [22]. Histological sections were examined using Zeiss Axioplan microscope (Carl Zeiss MicroImaging, Thornwood, NY) magnification 40×.

### Statistical analysis

Statistical analysis was carried out using statistical package for social sciences [23], statistical analysis system [24], and Levesque [25]. Means and standard errors were calculated using Windows Excel, and the obtained data were analyzed statistically using multifactorial analysis of variance (ANOVA) with the general linear model for all treated groups, age, and their interactions. Data were subjected to two-way ANOVA as variables were fitted as independent factors, the following equation was used:

Y_ijkl_=µ+G_i_+W_k_+GW_ik_+e_ijkl_

Where Y_ijkl_ is the examined variable, µ is the overall mean of the model, B_i_ is the effect of group, W_k_ is the effect of age in weeks, GW_ik_ is the interaction of group by age of bird, and e_ijkl_ is the error. Bivariate correlation coefficient was calculated to compare the influence of bacterial counts, immune organs’ weight, and IGs concentration on each other.

## Results

In Table-2, body weight showed significant increase (p<0.01) with the increase in *N. sativa* Linn. supplementation, especially in broilers fed on 4.2% and 5.6% *N. sativa* Linn. Weight gain and PI revealed a superiority in birds fed on 5.6% with significant differences (p<0.01) compared to the other treated groups and controls. Meanwhile, FCR revealed non-significant differences between all supplemented groups compared to the control.

**Table-2:** Performance traits (mean±SE) in different experimental groups supplemented with *N. sativa* Linn. in face of *E. coli* challenge.

*N. sativa* Linn.	Body weight (g)	Weight gain (g)	FCR (%)	Performance index
1.4%	971.767^c^±4.070	422.033^a^±6.554	1.439^b^±0.028	6.835^b^±0.181
2.8%	1055.230^b^±19.083	372.893^ab^±4.131	1.558^b^±0.013	6.655^b^±0.686
4.2%	1083.467^ab^±20.383	388.500^ab^±9.152	1.526^b^±0.121	7.082^ab^±0.336
5.6%	1118.363^a^±29.522	418.210^a^±6.439	1.306^b^±0.186	7.834^a^±0.254
Control	963.133^c^±2.316	330.900^b^±5.600	2.048^a^±0.115	5.443^c^±0.981
p value	0.001	0.010	0.001	0.001

Means carrying different superscripts in the same column are significantly different at p≤0.05 or highly significantly different at p<0.01. Means carrying the same superscripts in the same column are non-significantly different at p>0.05.*N. sativa = Nigella sativa*, *E. coli* = Escherichia coli, FCR = Feed conversion ratio

In Table-3, proteinogram revealed a highly significant increase (p<0.01) in total protein and albumin and a highly significant decrease (p<0.01) in globulin in broilers fed 5.6% *N. sativa* Linn. Liver enzymes (ALT, AST) and creatinine also showed a highly significant increase (p<0.01) in broilers fed 5.6% *N. sativa* Linn. On the contrary, urea revealed a significant increase in all treated groups from the normal control group with high significant differences (p<0.01).

**Table-3:** Biochemical parameters (mean±SE) in different experimental groups supplemented with *N. sativa* Linn. in the face of *E. coli* challenge.

*N. sativa* Linn.	Total protein (g/dl)	Albumin (g/dl)	Globulin (g/dl)	ALT (IU/L)	AST (IU/L)	Urea (mg/dl)	Creatinine (mg/dl)
1.4%	3.57^d^±0.017	1.87^d^±0.002	1.69^c^±0.025	27.18^b^±0.051	36.18^b^±0.098	79.26^b^±0.132	0.56^d^±0.012
2.8%	4.39^b^±0.008	3.17^b^±0.008	1.22^d^±0.014	26.91^c^±0.020	36.18^b^±0.154	75.12^c^±0.054	0.74^b^±0.003
4.2%	4.39^b^±0.014	2.37^c^±0.007	2.02^a^±0.013	25.71^d^±0.051	34.88^c^±0.103	73.62^d^±0.688	0.49^e^±0.006
5.6%	4.99^a^±0.012	3.87^a^±0.012	1.12^e^±0.030	27.41^a^±0.081	36.68^a^±0.075	75.72^c^±0.233	0.84^a^±0.018
Control	3.59^c^±0.007	1.87^d^±0.027	1.72^b^±0.018	27.21^b^±0.104	36.18^b^±0.132	81.62^a^±0.054	0.59^c^±0.025
p value	0.001	0.001	0.001	0.001	0.001	0.001	0.001

Means carrying different superscripts in the same column are significantly different at p≤0.05 or highly significantly different at p<0.01. Means carrying the same superscripts in the same column are non-significantly different at p>0.05. ALT=Alanine aminotransferase, AST=Aspartate aminotransferase, *N. sativa=Nigella sativa*, *E. coli*=*Escherichia coli*, SE=Standard error

IgG and IgA revealed a highly significant improvement in broilers fed 4.2% and 5.6% *N. sativa* Linn. with significant differences (p<0.01) from the other groups (Table-4), while IgM revealed non-significant differences among different groups. On the other hand, IGs revealed a significant (p<0.01) strong positive correlations between each other, and the increases in IgG and IgA are accompanied with an increase in IgM (Table-5).

**Table-4:** Immunoglobulin levels and logarithmic bacterial count (mean±SE) in different experimental groups supplemented with *N. sativa* Linn. in the face of *E. coli* challenge.

*N. sativa* Linn.	Immunoglobulin			Log bacterial count	
	
	IgG (mg/ml)	IgA (mg/ml)	IgM (mg/ml)	TBC (CFU/ml)	TEC (CFU/ml)
1.4%	1.359^a^±0.006	0.153^a^±0.003	0.204^a^±0.006	3.664^d^±0.005	3.990^a^±0.014
2.8%	1.195^b^±0.026	0.144^b^±0.085	0.200^a^±0.013	3.673^d^±0.006	3.760^b^±0.063
4.2%	1.340^a^±0.027	0.155^a^±0.007	0.206^a^±0.014	3.812^b^±0.011	3.113^d^±0.015
5.6%	1.194^b^±0.002	0.143^b^±0.008	0.199^a^±0.006	3.769^c^±0.006	2.299^e^±0.018
Control	1.318^a^±0.009	0.153^a^±0.002	0.204^a^±0.004	5.747^a^±0.002	3.502^c^±0.009
p value	0.001	0.001	0.662	0.001	0.001

Means carrying different superscripts in the same column are significantly different at p≤0.05 or highly significantly different at p<0.01. Means carrying the same superscripts in the same column are non-significantly different at p<0.05. *N. sativa=Nigella sativa*, *E. coli=Escherichia coli*, SE=Standard error, TBC=Total bacterial count, TEC=Total *Enterobacteriaceae* count, Ig=Immunoglobulin

**Table-5:** Correlation coefficient between immune organs’ weight, Ig levels with logarithmic TBC (above diagonal), and logarithmic TEC (below diagonal).

r	Log TBC	Thymus	Spleen	Bursa	IgG	IgA	IgM
Log TEC	1	0.331**	0.177*	0.010	0.266**	0.363**	0.257**
Thymus	0.092	1	0.853**	0.829**	0.170*	0.645**	0.148
Spleen	0.042	0.853**	1	0.831**	0.274**	0.721**	0.269**
Bursa	0.227**	0.829**	0.831**	1	0.033	0.527**	0.013
IgG	−0.474**	0.170*	0.274**	0.033	1	0.801**	0.990**
IgA	−0.200*	0.645**	0.721**	0.527**	0.801**	1	0.795**
IgM	−0.454**	0.148	0.269**	0.013	0.990**	0.795**	1

** Correlation is highly significant (p<0.01).

* Correlation is significant (p<0.05).

^NS^Correlation is non-significant (p>0.05). TBC = Total bacterial count, TEC = Total *Enterobacteriaceae* count, Ig = Immunoglobulin

In Table-4, TBC and TEC showed highly significant decline (p<0.01) in broilers fed 5.6% *N. sativa* Linn. compared to the other groups. TBC in Table-5 revealed a highly significant weak positive correlations (p<0.01) with all of thymus, IgG, IgA, and IgM concentrations. On the other hand, TEC revealed significant intermediate negative correlations (p<0.01) with IgG and IgM. A significant weak negative correlation was detected between TEC and IgA. Furthermore, a highly significant weak positive correlations (p<0.01) was detected between TEC and bursa. In Table-6, immune organs (thymus, spleen, and bursa) revealed a synchronized highly significant improvement (p<0.01) in broilers fed 5.6% *N. sativa* Linn. compared to the other groups.

**Table-6:** Immune organs’ weight (mean±SE) in different experimental groups supplemented with *N. sativa* Linn. in the face of *E. coli* challenge.

*N. sativa* Linn	Thymus (g/kg)	Spleen (g/kg)	Bursa (g/kg)
1.4%	2.494^c^±0.008	2.495^c^±0.045	2.546^a^±0.036
2.8%	2.415^d^±0.105	2.586^b^±0.021	1.793^d^±0.081
4.2%	2.900^a^±0.027	2.460^c^±0.101	2.014^c^±0.010
5.6%	2.743^b^±0.018	2.760^a^±0.054	2.334^b^±0.011
Control	2.791^b^±0.063	2.211^d^±0.081	1.674^e^±0.041
p value	0.001	0.001	0.001

Means carrying different superscripts in the same column are significantly different at p≤0.05 or highly significantly different at p<0.01. Means carrying the same superscripts in the same column are non-significantly different at P>0.05. *N. sativa*=*Nigella sativa*, *E. coli=Escherichia coli*, SE=Standard error

**Figure-1:**
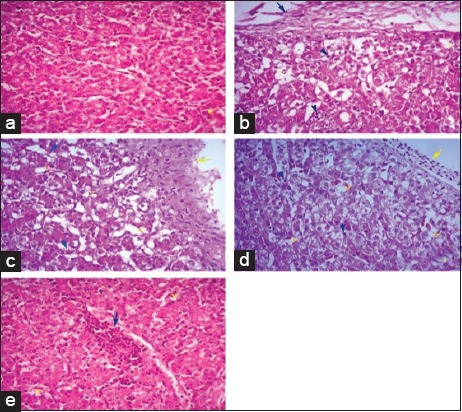
Histopathological examination of the liver in different broilers groups fed on different concentrations of *Nigella sativa* Linn. (a) Liver of control broilers. (b) Liver of broilers fed *N. sativa* Linn. 1.4%. (c) Liver of broilers fed *N. sativa* Linn. 2.8%. (d) Liver of broilers fed *N. sativa* Linn. 4.2%. (e) Liver of broilers fed N. sativa Linn. 5.6% (H and E, 40×).

**Figure-2:**
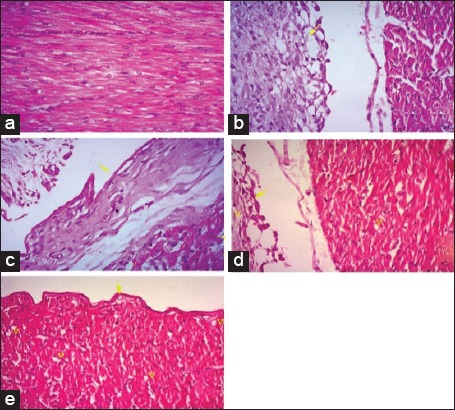
Histopathological examination of the heart in different broilers groups fed on different concentrations of *Nigella sativa* Linn. (a) Heart of control broilers. (b) Heart of broilers fed *N. sativa* Linn. 1.4%. (c) Heart of broilers fed *N. sativa* Linn. 2.8%. (d) Heart of broilers fed *N. sativa* Linn. 4.2%. (e) Heart of broilers fed *N. sativa* Linn. 5.6% (H and E, 40×).

**Figure-3:**
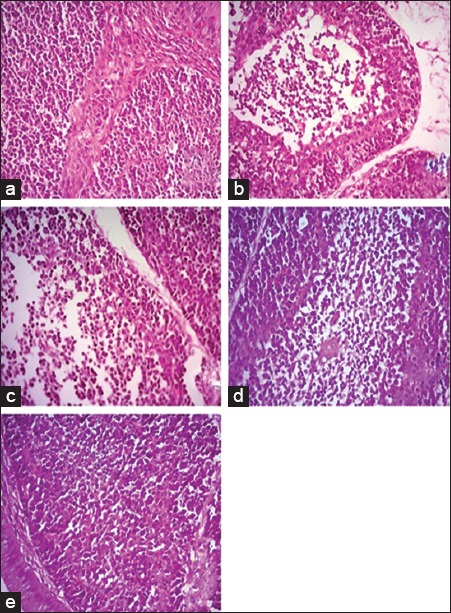
Histopathological examination of the Bursa in different broilers groups fed on different concentrations of *Nigella sativa* Linn. (a) Bursa of control broilers. (b) Bursa of broilers fed *N. sativa* Linn. 1.4%. (c) Bursa of broilers fed *N. sativa* Linn. 2.8%. (d) Bursa of broilers fed *N. sativa* Linn. 4.2%. (e) Bursa of broilers fed *N. sativa* Linn. 5.6% (H and E, 40×).

**Figure-4:**
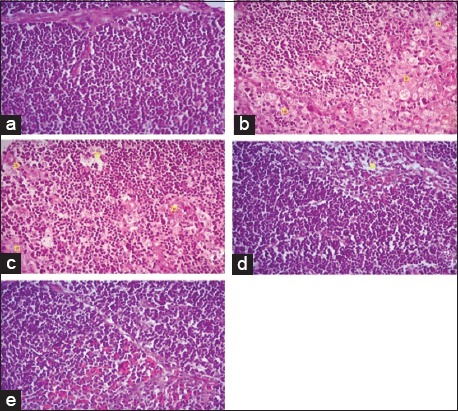
Histopathological examination of the thymus in different broilers groups fed on different concentrations of *Nigella sativa* Linn. (a) Thymus of control broilers. (b) Thymus of broilers fed *N. sativa* Linn. 1.4%. (c) Thymus of broilers fed *N. sativa* Linn. 2.8%. (D) Thymus of broilers fed *N. sativa* Linn. 4.2%. (e) Thymus of broilers fed *N. sativa* Linn. 5.6% (H and E, 40×).

**Figure-5:**
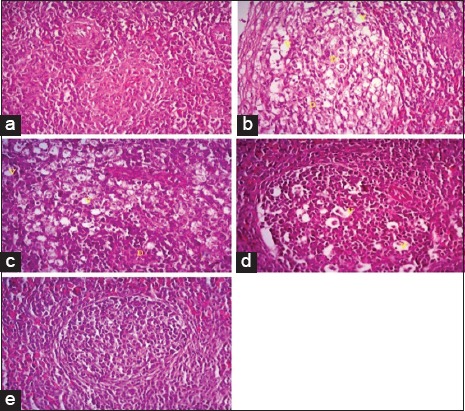
Histopathological examination of the spleen in different broilers groups fed on different concentrations of *Nigella sativa* Linn. (a) Spleen of control broilers. (b) Spleen of broilers fed *N. sativa* Linn. 1.4%. (c) Spleen of broilers fed *N. sativa* Linn. 2.8%. (d) Spleen of broilers fed *N. sativa* Linn. 4.2%. (e) Spleen of broilers fed *N. sativa* Linn. 5.6% (H and E, 40×).

Histopathological section of liver from broilers fed on *N. sativa* Linn. 1.4% and 2.8% as shown in Figure-1b and c revealed severe perihepatitis, severe leukocytic infiltrations, and hydropic degeneration in majority of hepatocytes compared to normal microscopic picture of liver in Figure-1a. Heart in broilers fed on 1.4% and 2.8% *N. sativa* Linn. (Figure-2b and c) showed severe pericarditis with partial extension to myocardium resulting in myocarditis compared to normal histological picture of heart (Figure-2a). Some of the myocardial cells showed vacuolated cytoplasm. Meanwhile, bursa, thymus, and spleen of broilers fed on 1.4% and 2.8% *N. sativa* Linn. (Figures-3b and c, 4b and c, 5b and c) revealed severe lymphoid depletion compared to normal histological pictures (Figures-3a, 4a and 5a).

The examination of liver from broilers fed on *N. sativa* Linn. 4.2% in Figure-1d showed a moderate degree of perihepatitis with vacuolated cytoplasm, in the majority of hepatocytes, the vacuoles surrounded with irregular boundaries indicating hydropic degeneration with leukocytic infiltration compared to normal microscopic picture of liver (Figure-1a). Heart in broilers fed on *N. sativa* Linn. 4.2% (Figure-2d) revealed moderate pericarditis, and some of myocardial cells showed vacuolated cytoplasm compared to normal histological picture in Figure-2a. Spleen, bursa, and thymus of broilers fed on *N. sativa* Linn. 4.2% (Figures-3d, 4d and 5d) showed mild lymphoid depletion with severe vacuolation compared to normal microscopic pictures (Figures-3a, 4a and 5a).

Liver of broilers fed on *N. sativa* Linn. 5.6% in Figure-1e showed pronounced improvement of hepatocytes with no proliferation of fibroblasts, and only mild vacuolar degeneration of hepatocytes and mild leukocytic infiltration were observed compared to normal microscopic picture of liver (Figure-1a). Heart of broilers fed on 5.6% *N. sativa* Linn (Figure-2e) showed mild pericarditis with a mild degree of hydropic degeneration in some myocardial cells compared to normal microscopic appearance (Figure-2a). Bursa, thymus, and spleen of broilers fed on *N. sativa* Linn. 5.6% (Figures-3e, 4e and 5e) showed lymphoid hyperplasia compared to normal histological picture in Figures-3a, 4a and 5a.

## Discussion

*N. sativa* Linn. was reported as growth promoter and significant source of essential nutritive substances according to the study of Attia *et al*. [1,8,9], El-Deek *et al*. [10], and Benhelima *et al*. [26], including about 26.7% protein, 33.2% carbohydrate, and 38.7% fat. These nutrients contributed a great enhancement in performance traits, especially body weight and weight gain, after supplementation in a rate of 1 and 1.5% for 42 days as reported by EL-Shoukary *et al*. [27], these results were in favor of our results which concluded that *N. sativa* Linn. supplementation at rate of 4.2% and 5.6% was successfully able to enhance the performance traits.

Meanwhile, FCR in the current study revealed non-significant differences between all supplemented groups compared to the control. Controversy results were documented by Boka *et al*. [28], who found a great enhancement in FCR in broilers supplemented with 0, 1, 2, and 3% *N. sativa* Linn. seeds, and the best FCR was recorded in broilers fed on 2% *N. sativa* Linn. Controversy results were documented by Jahan *et al*. [29], who found that FCR was improved using 1.5% *N. sativa* Linn. seeds at the early age of 14 days old.

Others indicated that *N. sativa* Linn. supplementation at rates of 0.25%, 0.75%, 1%, and 2% had undesirable effects on performance and carcass quality as recorded by Majeed *et al*. [30] and Nasir and Grashorn [31], the same conclusions were found in our study, especially, that lower concentrations of *N. sativa* Linn. (1.4%, 2.8%) were not able to exert these performance enhancement and immune-stimulant effects. Meanwhile, Marian H. Ghaly *et al*. [32] found that addition of *N. sativa* and their combinations for long periods could not alter liver and kidney histology and physiology and increased liver weight and dressing percentage.

*N. sativa* Linn., in our study, resulted in a significant increase in total protein and albumin and significant decrease in globulin in G4 in agreement with the findings of AL-Beitawi *et al*. [33], who found that by the addition of 2% *N. sativa* Linn. seeds in broiler diet resulted in increased total plasma protein. The present study disagreed with the findings of Salam *et al*. [34], who found that feeding 20, 40, and 60 g/kg *N. sativa* Linn. seeds in broilers diet did not significantly affect on physical properties of blood. Azeem *et al*. [35] showed that supplementation of broilers diet with *N. sativa* Linn. 1 or 3% strengthened the immunity by preventing liver damage and decreasing serum phospholipids and cholesterol. Furthermore, Hermes *et al*. [36] found a role of *N. sativa* Linn. in activating the function of the liver without any toxic effect on the liver or kidney; they reported unaltered liver enzyme activity in contrast to our results which is supported with the study of Sogut *et al*. [37], who showed that the *N. sativa* Linn. decreased the hepatic liver peroxidation and increased the activities of several enzymes such as glutathione-S-transferase, catalase, myeloperoxidase, and adenosine deaminase all of which resulted in decreased oxidative stress on the liver using 3, 5, and 7% *N. sativa* Linn.

Our results revealed significant improvement in IgG and IgA of birds fed on *N. sativa* Linn. 4.2% and 5.6% and non-significant changes in IgM in all treated groups and control. A highly significant strong positive correlations was detected in IGs pattern. The same obtained with Al-Mufarrej [38], who investigated the immune-responsiveness and performance of broiler chickens fed *N. sativa* Linn. powder, his study showed that dietary supplement of *N. sativa* Linn. seeds at the level of 1% or 1.4% would enhance immune responsiveness in broiler chickens.

The synchronized highly significant improvement of thymus, spleen, and bursa in birds fed on *N. sativa* Linn. 5.6% was supported by the results showed by Umar *et al*. [39], who reported an enhanced immune responsiveness and reduced pathogenicity of Avian Influenza H9N2 in birds supplemented with *N. sativa* Linn. 3%.

*N. sativa* Linn. was also able to increase the broilers resistance with effective limitation for the propagation of *Enterobacteriaceae* as reported by Erener *et al*. [40], these finding supported our finding for the significant decline of both total and *Enterobacteriaceae* counts from the broilers’ intestine. The present study also revealed a significant increase in IG concentration (IgG and IgA) with great enhancement of the histopathological picture of immune organs (thymus, bursa, and spleen). These findings were supported by those declared by Altunoglu *et al*. [41] and Shewita and Taha [42]. From the previous results concerning IGs, immune organs, and bacterial count, we could prove the protective and immune-stimulation effect of *N. sativa* Linn.

*E. coli* infection in birds contributed colibacillosis and subsequent morbidity and mortality, with heavy economic losses in poultry industry. *E. coli* infection in broilers usually opened a gate for secondary infection with other microorganisms through lowering the bird’s resistance causing severe illness and deaths in birds. Colibacillosis usually characterized by septicemia in acute stage resulting in death and pericarditis, airsacculitis, and perihepatitis in the subacute stage [43]. *E. coli* infection was clearly known to contribute marked gross and microscopic bursal lesions causing bursal atrophy that subsequently resulted in transient humeral immunosuppression [44].

*N. sativa* Linn. has been reported to exert many biological activities as immune-stimulant, respiratory-stimulant, antihypertensive, antidiabetic, analgesic, anti-inflammatory, anti-ulcerogenic, antibacterial, antifungal, anthelmintic, and antitumor actions [1,45,46]. *N. sativa* Linn. was found to have an antimicrobial activity on *E. coli*, *Bacillus subtilis*, *Streptococcus faecalis*, *Staphylococcus aureus*, *Pseudomonas aeruginosa*, and *Candida albicans* [47,48].

Our result revealed that *N. sativa* Linn. 1.4% and 2.8% showed severe perihepatitis and severe pericarditis with partial extension to myocardium, resulting in myocarditis. Furthermore, bursa, thymus, and spleen revealed severe lymphoid depletion. The histopathological picture of these concentrations improves the inability to perform a protective action against *E. coli*. These lesions were in consistent with the study of Bopp *et al*. [49], who found that *E. coli* infection in broilers prevailed at 3-12 weeks, and frequently followed by generalized septicemia, perihepatitis, and pericariditis. Nakamura *et al*. [50] also mentioned that *E. coli* infection contributed a severe damage in the immune systems of broilers including lymphocyte depletion in both bursa and thymus.

*N. sativa* Linn. was reported to have anti-inflammatory [51], renal-protective [52], hepatoprotective [53], and immune-potentiating [54] properties. *N. sativa* Linn. also had an antibacterial actions against a wide range of microorganisms [55]. Our results revealed that broilers feed on *N. sativa* Linn. 4.2% showed moderate perihepatitis and moderate pericarditis. Spleen, bursa, and thymus showed mild lymphoid depletion. While feeding on *N. sativa* Linn., 5.6% showed pronounced improvement of hepatocytes with no proliferation of fibroblasts, mild pericarditis. Bursa, thymus, and spleen showed lymphoid hyperplasia.

Histopathological examination in our study emphasizes the improvement in the organs’ pictures on feeding higher level of *N. sativa* Linn., and this is may be due to its antimicrobial properties, which could decrease the harmful effect after challenge with *E. coli*. The present findings are in contrast to that of Marian *et al*. [32], who revealed that the histopathological examination, after feeding on *N. sativa* Linn. oil (at a dosage rate of 2 ml/kg basal diet) for 6 weeks in 1-day-old broilers, at day 21, and day 42 of broilers age, the liver and kidney sections showed degeneration, he reported that it might be due to the toxic effects of *N. sativa* Linn.

## Conclusion

*N. sativa* Linn. supplementation at the rate of 4.2% and 5.6% in broilers can be used efficiently as growth promoters to enhance the performance traits including BWG, FCR, and PI and as an immune-modulatory agent to increase the birds’ resistance against many diseases including *E. coli* infection.

## Authors’ Contributions

ESS designed the experimental design, prepared, supervised, and assisted in each step during the experiment. RTH assisted in laboratory work and conducted the histopathological examination. AA assisted in laboratory work and in writing of the manuscript. All authors read and approved the final manuscript.
